# Path Planning Trends for Autonomous Mobile Robot Navigation: A Review

**DOI:** 10.3390/s25041206

**Published:** 2025-02-16

**Authors:** Yuexia Tang, Muhammad Aizzat Zakaria, Maryam Younas

**Affiliations:** 1 Faculty of Manufacturing and Mechatronic Engineering Technology, Universiti Malaysia Pahang AL-Sultan Abdullah, Pekan 26600, Pahang Darul Makmur, Malaysia; 2 Intelligent Manufacturing College, Nanning University, Nanning 530299, China; 3 Autonomous Vehicle Laboratory, Centre for Automotive Engineering, Universiti Malaysia Pahang AL-Sultan Abdullah, Pekan 26600, Pahang Darul Makmur, Malaysia; 4 Centre for Advanced Industrial Technology, Universiti Malaysia Pahang AL-Sultan Abdullah, Pekan 26600, Pahang Darul Makmur, Malaysia

**Keywords:** autonomous driving, path planning algorithm, global path planning, local path planning, intelligent planning algorithm

## Abstract

With the development of robotics technology, there is a growing demand for robots to perform path planning autonomously. Therefore, rapidly and safely planning travel routes has become an important research direction for autonomous mobile robots. This paper elaborates on traditional path-planning algorithms and the limitations of these algorithms in practical applications. Meanwhile, in response to these limitations, it reviews the current research status of recent improvements to these traditional algorithms. The results indicate that these improved path-planning algorithms perform well in tests or practical applications, and multi-algorithm fusion for path planning outperforms single-algorithm path planning.

## 1. Introduction

Autonomous mobile robotics technology plays a crucial role in enhancing operational safety, optimizing task execution efficiency, reducing operational errors, and mitigating environmental burdens. By leveraging high-precision environmental perception, intelligent decision-making, and path planning technologies, it enables autonomous mobile robots to navigate independently, becoming a core component of future intelligent operational systems.

The core of autonomous mobile robotics technology encompasses four main aspects: environmental perception for analyzing the surrounding environment [1]; behavioral decision-making for determining the robot’s action plans [2]; path planning for mapping out the robot’s movement trajectories [3]; motion control for governing the specific movements of the robot [4].

**Figure 1:**
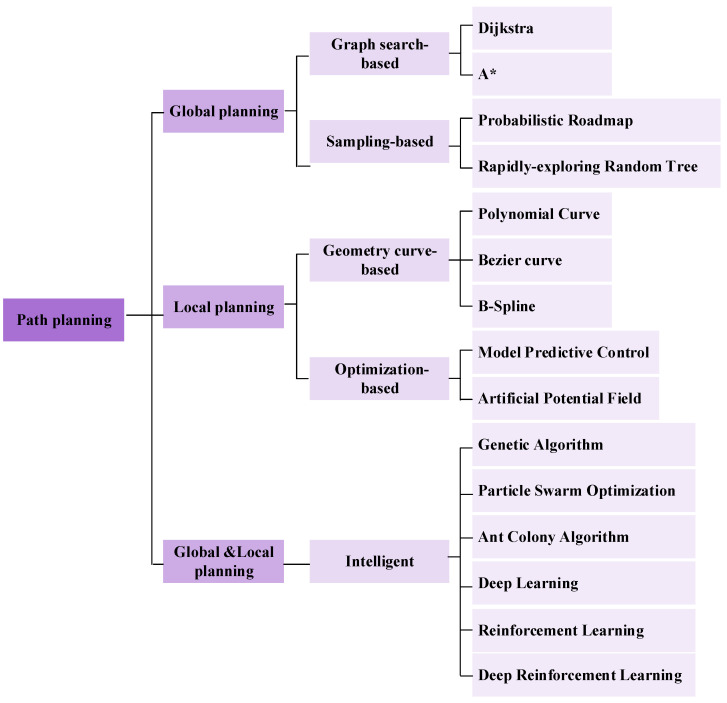
The classification and common algorithms of path planning.

Path planning is a crucial component of autonomous mobile robotics technology. It ensures that the robot reaches its destination smoothly by designing the optimal movement path [5]. Path planning can be divided into global planning and local planning. Global planning is the overall path design from the starting point to the destination, which usually generates a complete path based on map information and known environmental data [6]. Local planning, on the basis of the globally planned path, performs short-term path planning according to the current environmental state of the vehicle or robot [7]. It mainly focuses on issues such as dynamic obstacles, real-time obstacle avoidance, and path smoothing [8]. Figure 1 shows the classification and common algorithms of path planning.

For path planning, scholars around the world have proposed a variety of algorithms. This paper provides an analysis and outlook on the current research status of path-planning technology for autonomous mobile robots. The 15 path planning algorithms shown in Figure 1 will be introduced in the subsequent content. Section 2 introduces graph search-based algorithms. Section 3 discusses sampling-based planning algorithms. Section 4 covers geometry curve-based planning algorithms. Section 5 presents optimization-based planning algorithms. Section 6 introduces intelligent algorithms. Finally, Section 7 provides a conclusion.

## 2. Graph Search-Based Planning Algorithms

In graph theory, a graph is a structure composed of nodes (or vertices) and edges that connect these nodes. Graph-based search planning algorithms use specific search strategies within such a graph structure to find the optimal or feasible path from a starting node to a goal node. Common algorithms are Dijkstra and A*.

### 2.1. Dijkstra Algorithm

**Figure 2:**
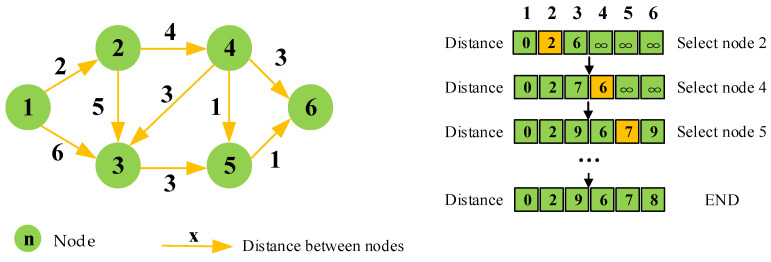
The process of the Dijkstra algorithm.

Dijkstra’s algorithm is a classical graph search algorithm that was proposed by the Dutch computer scientist Edsger W. Dijkstra in 1956 [9]. As shown in Figure 2, during its execution, Dijkstra’s algorithm maintains a distance table that records the current shortest path lengths from the starting node to each node. From the starting node, the algorithm gradually selects the node with the smallest distance for expansion, updates the distances of its neighboring nodes, and adds these nodes to the set of nodes with known shortest paths. By iterating this process until all nodes are included in the set, the algorithm finds the shortest path from the starting node to all other nodes [10].

The Dijkstra algorithm is widely used in various shortest-path problems. However, it cannot handle negative weight edges and responds slowly to dynamically changing environments. Therefore, many scholars and researchers have made in-depth explorations of and improvements to the Dijkstra algorithm.

Bing et al. [11] proposed two methods to reduce the amount of calculation. First, when calculating the minimum distance between a certain node and the starting node, the algorithm takes into account the distance between that node and the goal point, guiding the exploration in a general direction towards the destination. Second, the ellipse model was used to limit the search scope, thereby reducing unnecessary calculations. The traditional Dijkstra algorithm usually uses a four-neighborhood search in a grid graph. Sun et al. [12] proposed the octagonal search method, which allows searching from a free grid in eight directions: West, Southwest, North, Northwest, East, Northeast, South, and Southeast. In this way, a shorter and smoother path can be planned, reducing the number of turns and turning angles.

### 2.2. A* Algorithm

**Figure 3:**
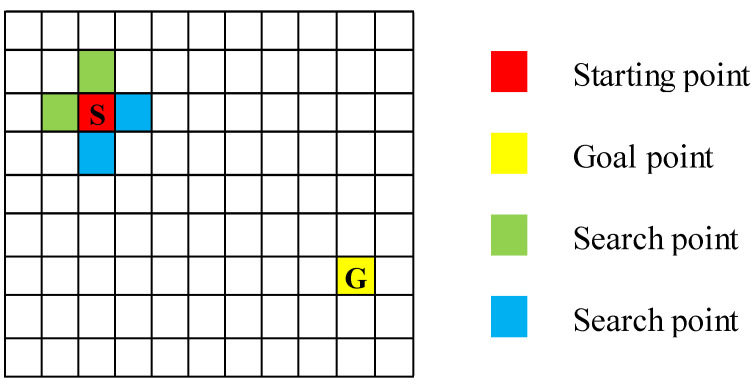
The search direction of Dijkstra and A*.

The A* algorithm is an extension of Dijkstra’s algorithm [13], which introduces a heuristic function h(n) to estimate the cost from the current node to the target node [14], thereby accelerating the pathfinding process [15]. As shown in Figure 3, Dijkstra’s algorithm explores all reachable nodes in the graph (represented by blue and green nodes in the figure), whereas A* takes into account the direction of the goal point and searches more selectively, focusing on the path indicated by the blue color in the figure.

Although the A* algorithm is effective in pathfinding, it also has some limitations, such as slow planning speed, numerous redundant nodes, high collision risk, and poor path smoothness. These issues have prompted researchers to develop various algorithms to optimize the A* algorithm, aiming to improve the efficiency, stability, and overall performance of path-planning tasks. Common approaches include improving the heuristic function, introducing bidirectional search, performing path smoothing, adopting hybrid algorithms, and extending obstacle distances, as shown in Table 1.

In addition to the aforementioned common methods, some scholars have also proposed other improvement methods. Wang et al. [26], based on the A* algorithm, combined it with the Jump Point Search (JPS) algorithm by replacing the expanded nodes in the A* algorithm with jump points to reduce the computational load. Second, they applied a pruning algorithm to prune the obtained path, further eliminating unnecessary nodes from the path. Finally, they smoothed the path using quadratic Bezier curves. Li et al. [27] proposed an adaptive step size algorithm that dynamically adjusts based on obstacle distribution, reducing node expansions. Then, they used cubic Bezier curves to smooth the path. The algorithm also integrates DWA: improved A* for global planning, DWA for local planning, and real-time obstacle avoidance, addressing the limitations of A* in dealing with dynamic obstacles. Li et al. [28] designed an improved A* algorithm based on graph preprocessing. First, they used an enhanced convex decomposition method based on Maklink to decompose the free space on the map into multiple polygonal regions. Then, each region was encoded as a feature node according to the A* algorithm. Last, based on the principles of the A* algorithm, the optimal regional pathway was identified, resulting in a globally optimal path solution.

### 2.3. Summary

Both A* and Dijkstra are classic algorithms in graph theory for finding the shortest path. Table 2 presents a comparison of these two algorithms.

The A* algorithm often demonstrates higher efficiency in most practical applications. However, the Dijkstra algorithm exhibits its unique advantages in certain specific scenarios due to its stability and simplicity. After being optimized through the aforementioned methods, these two algorithms have improved their efficiency or path quality to some extent, but they also introduce additional computations, which may increase the complexity and computational cost of the algorithms.

## 3. Sampling-Based Planning Algorithms

These algorithms construct paths or roadmaps by randomly sampling the planning space, thereby discovering feasible paths from the start to the goal. By randomly sampling the search space, these algorithms progressively explore and identify feasible paths, eliminating the need for an exhaustive search of the entire space. This significantly enhances efficiency.

### 3.1. Probabilistic Roadmap (PRM)

RPM constructs a graph, referred to as a roadmap, which represents feasible paths. This is accomplished by randomly sampling points, connecting them, and then searching for the optimal or feasible path from the start to the goal within this graph [29].

**Figure 4:**
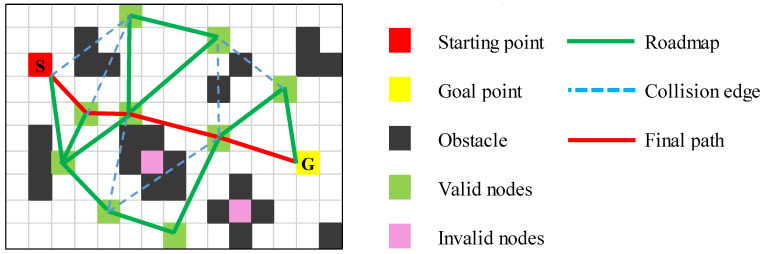
PRM algorithm.

The PRM algorithm splits path planning into two phases: learning and query [30]. As shown in Figure 4, in the learning phase, nodes are randomly sampled in the configuration space, and invalid nodes (intersecting obstacles) are removed. Valid nodes become graph vertices, and collision-free paths between them form edges, constructing a roadmap of the free space. During the query phase, traditional graph search algorithms (e.g., Dijkstra, A*) are applied to this roadmap to find the optimal path from start to goal [31].

However, the traditional PRM exhibits several limitations, including inefficiency, suboptimal performance in dynamic environments, and challenges in handling narrow passages. To mitigate these shortcomings, a range of enhancements and hybrid approaches have been developed.

#### 3.1.1. For Low Sampling Efficiency

Li et al. [32] optimized sampling distribution using a pseudo-random strategy and spatial axis. They reduced collision detections with a two-way method and improved roadmap efficiency by setting a connection threshold. Ma et al. [33] proposed a bidirectional search strategy, which involves simultaneously searching from both the start and goal points, alternating between forward and reverse searches. This approach enables faster identification of overlapping nodes in the middle, thereby reducing unnecessary node connections, forming a complete path, and improving the convergence speed of the algorithm. Liu et al. [34] first constructed the topological skeleton of the workspace. Subsequently, guided by this skeleton, they dynamically adjusted the sampling regions, prioritizing the coverage and connection of critical areas, especially narrow passages. By ensuring that each sampling region focused on expanding specific local connection components and introducing additional connection steps to merge different components, they effectively enhanced the local connectivity and overall coverage of the roadmap. In the relative motion mode, Ahmed et al. [35] divided the planning domain into multiple subdomains with overlapping boundaries. Path planning was carried out separately within each subdomain, considering only the nodes and obstacles within the current subdomain. The paths from each subdomain were then pieced together to form a complete global path.

#### 3.1.2. For Narrow Passages

Ravankar et al. [36] created a potential field map based on obstacle information, dividing the map into high-potential and low-potential regions. They placed more nodes in high-potential regions (near obstacles) and fewer nodes in low-potential regions to optimize the distribution of sampling points. This approach solved the connectivity issue of PRM in narrow passages and reduced computational costs. Li et al. [37] proposed reducing the sampling range to an elliptical area focused on the start and end points, which makes the sampling points more concentrated and improves sampling efficiency. Within the focused sampling range, the sampling density is dynamically adjusted according to the terrain and obstacle distribution. Sampling points are appropriately increased near obstacles or within narrow passages to ensure that the path map remains connected in these areas. Cao et al. [38] proposed a novel sampling strategy. First, they conducted adaptive random sampling in the global map to ensure that sufficient sampling points were generated around obstacles. Subsequently, they selected sampling points inside or near obstacles and performed uniform sampling within a certain distance (which could be dynamically adjusted) around these points. If a sampling point fell within the free space, it was retained; otherwise, resampling was carried out.

#### 3.1.3. Hybrid Algorithm

As shown in Table 3, combining PRM with other algorithms is expected to improve the efficiency of path planning, potentially shortening path lengths and enhancing the avoidance of dynamic obstacles to some extent.

### 3.2. Rapidly-Exploring Random Tree (RRT)

**Figure 5:**
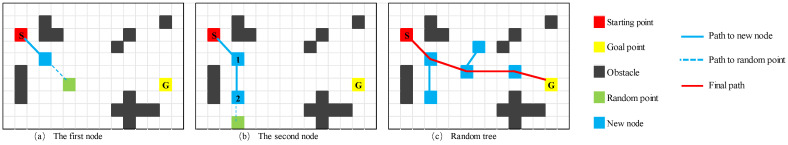
The path generation process of RRT.

The RRT algorithm is a global path-planning method utilizing sampling [42]. As shown in Figure 5a, set the starting point as the root node of the random tree. Randomly generate a sampling point (green) and identify the node in the random tree with the shortest distance to it. Take a step from this nearest node towards the sampling point to create a new node (blue). Repeat these steps as illustrated in Figure 5b. Continue until the destination is included within the random tree or the maximum number of iterations is reached, as depicted in Figure 5c.

The RRT algorithm has been widely applied in the field of path planning due to its ability to explore large spaces quickly [43]. However, the basic RRT algorithm may sometimes generate non-optimal paths, and its convergence speed can be relatively slow. To address these shortcomings, researchers have developed various improved algorithms.

Zhang et al. [44] compared the distance between the new node and both the nearest node on the random tree and its parent node. If the new node is closer to its parent than to the nearest node, it is considered to have regressive properties and should be filtered out to avoid repeated searches. Meanwhile, a state value is maintained for each node, which is dynamically adjusted based on whether the node’s child nodes result in collisions. If a node has a high collision probability, its future probability of being expanded is reduced, thereby guiding the random tree to avoid obstacles. Guo et al. [45] proposed the HDM-RRT algorithm, which generates a Collision Risk Map (CR-Map) by integrating vector layer data from high-definition maps and sensor perception data. Reference points are selected using a Gaussian distribution, and the sampling range and density are then adjusted based on the collision risk coefficients in the CR-Map. Finally, a cost function and a rewiring process are designed to optimize node selection. More research is presented in Table 4.

### 3.3. Summary

PRM and RRT are two classic sampling-based path planning algorithms, each with its own advantages and disadvantages, suitable for different scenarios. As can be seen from Table 5, PRM performs relatively well in global path planning in static environments, especially in high-dimensional spaces, where it can generate global paths relatively efficiently and has a comparative advantage in multiple query scenarios. On the other hand, RRT is more suitable for dynamic environments and applications requiring real-time responses, as it can quickly find feasible paths through incremental search, although the generated paths may require further smoothing and optimization.

After being optimized by the aforementioned methods, these two algorithms have overcome their limitations to some extent. However, the introduction of additional information and processing steps may increase the computational complexity of the algorithms, potentially limiting their application in real-time scenarios. Furthermore, many improvement methods rely on specific parameter settings, which may affect the performance and generality of the algorithms.

## 4. Geometry Curve-Based Planning Algorithms

Path planning algorithms based on geometric curves generate paths using specific geometric curves, aiming to create smooth and continuous routes for vehicles or robots to ensure safe driving and enhance ride comfort. Common geometric curve-based path-planning algorithms include polynomial curves, Bezier curves, and B-spline curves.

### 4.1. Polynomial Curve

**Figure 6:**
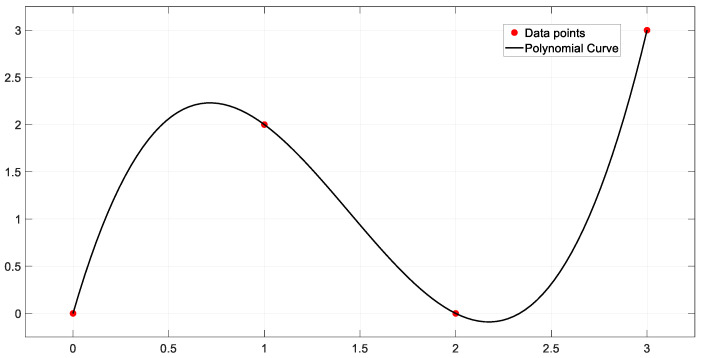
Polynomial Curve.

As shown in Figure 6, a polynomial curve is a smooth curve based on a polynomial equation, with the core idea of using polynomial functions to describe the motion trajectory of a vehicle or robot. In the field of automatic driving, polynomial curves are widely used to generate smooth and continuous driving paths. By adjusting the degree of the polynomial, the complexity and accuracy of the path can be controlled. The shape of the polynomial curve can be altered by adjusting its coefficients or the data points along the path [51].

Li et al. [52] proposed a three-stage curve interpolation method for parking path construction. First, a clothoid curve ensures a smooth curvature transition between the parking endpoint and the start of the arc. Next, an arc provides the required turning curvature. Finally, a quintic polynomial connects the arc endpoint to the parking start point, ensuring zero curvature at both ends and satisfying vehicle kinematic constraints. Zhang et al. [53] defined trajectory dimensions for scenarios such as car-following, lane-changing, and parking. They selected polynomial degrees based on vehicle dynamics and comfort. For each dimension, they established polynomial equations and solved for the coefficients using quadratic programming, objective functions, and constraints to generate the trajectory. Boryga et al. [54] proposed a method for optimizing the movement paths of agricultural machinery. The researchers analyzed four types of paths designed using polynomial transition curves (3rd, 5th, 7th, and 9th degree) and compared them in terms of their curvature, acceleration, and jerk. The simulation results showed that the path described by a cubic polynomial had the best performance. Huang et al. [55] designed smooth automatic parking paths using sixth-degree polynomials and easement curves, improving the quality of the vehicle’s parking experience. Vu et al. [56] proposed three methods. First, they used a quintic polynomial for position changes, but this method may cause large steering angles in short distances. Second, they proposed a new quartic polynomial method that decomposes dynamics into four variables, but it has non-smooth velocity changes. Third, they used symmetric polynomial functions to obtain smooth, symmetric trajectories with reduced tracking errors. The third method is more suitable for automatic parking.

### 4.2. Bezier Curve

**Figure 7:**
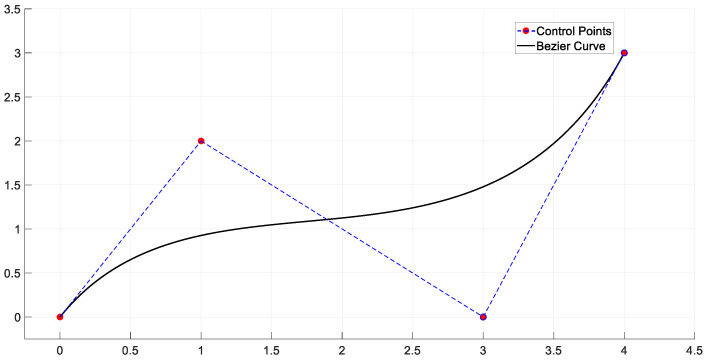
Bezier Curve.

Bezier curve determines its shape by defining the start point, endpoint, and one or more control points, as shown in Figure 7. By adjusting the position of the control points, the shape of the curve can be flexibly changed to generate a smooth path [57,58].

Zheng et al. [59] proposed a local trajectory planning algorithm using quartic Bézier curves, where the trajectory’s start and end serve as the first and last control points. Intermediate control points are optimized through mathematical derivation. The algorithm generates trajectory candidates by adjusting control point positions and iteratively finds the optimal trajectory meeting objectives and constraints using the SQP algorithm. Vinayak et al. [60] proposed a new algorithm for finding control points of Bézier curves. The algorithm randomly selects a series of points along the predefined path, which are key positions that the Bézier curve must pass through. By extending the Bézier curve’s mathematical expression and forming a system of equations, the control points are computed by solving these equations. Adjusting the parameters of these selected points alters the shape of the Bézier curve. Bae et al. [61] used Quintic Bézier curves to generate multiple possible lane-change trajectories. The optimal trajectory is then selected based on the user-defined lateral acceleration limits. If the initially generated trajectory does not meet the lateral acceleration constraints, the algorithm dynamically adjusts the control points and regenerates the trajectory until one that satisfies the conditions is found. Li et al. [62] designed a Quintic curve for lane changes with a leading vehicle. They defined boundaries for quick adaptation and comfort, then established a multi-objective optimization function, considering initial curvature, maximum curvature, path angle, and lane change time. Finally, they solved for control points to shape the optimal path.

### 4.3. B-Spline

**Figure 8:**
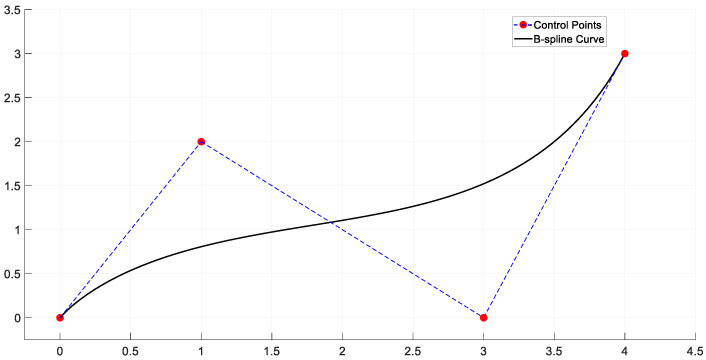
B-Spline.

The B-Spline is constructed from a series of control points and basis functions. It generates a smooth curve through the weighted average of these control points, as shown in Figure 8 [63]. B-spline curves offer local control, which means that modifying a control point only affects a local region of the curve without altering the entire curve [64].

Cao et al. [65] proposed a B-spline curve-based path-planning method for roundabout scenarios. The algorithm collects data points from the midpoint of the yield line at the entrance and every one-eighth section of the roundabout to define the B-spline knot vector. The control points are solved using linear equations, and a uniform B-spline interpolation connects the start, end, and control points to generate a smooth, continuous path. Wang et al. [66] assessed the risk of obstacles by analyzing parameters such as their size, type, speed, and relative position to the vehicle. Based on the risk assessment results, they considered road characteristics and vehicle dynamic constraints and used cubic quasi-uniform B-spline curves for path planning. By selecting and adjusting control points, they generated smooth paths. Cao et al. [24] proposed a dynamic path-planning method for indoor robots, fusing B-spline curves with an Improved Anytime Repairing A* (BS-IARA*) algorithm. It introduces adaptive expansion factors and dynamic search steps to address issues in traditional ARA*, such as low expansion factor preference, efficiency sensitivity, and uneven paths. By smoothing the initially planned path using B-spline curves. Zhang et al. [67] considered the vehicle’s motion characteristics during the lane-changing process and divided it into four stages: torsion angle, approach, angle closure, and adjustment. They placed control points at key positions in each stage and adjusted their locations to ensure that the path could avoid obstacles. Using the optimized control points and the B-spline curve equation, they generated a complete obstacle avoidance path.

### 4.4. Summary

Polynomial curves, Bezier curves, and B-spline curves are all used in path planning and curve design to generate smooth trajectories, but they differ in terms of structure, computational complexity, and application scenarios, as shown in Table 6.

In summary, polynomial curves, Bezier curves, and B-splines each exhibit unique application scenarios and advantages in path planning. The specific choice of which curve to apply in practice depends on the computational complexity, smoothness requirements, real-time demands, and computational resource constraints of the desired path. These algorithms have indeed made significant progress in trajectory generation and optimization after being optimized. However, in practical applications, they may still face relatively high computational complexity, complex parameter settings, and certain limitations in scenario adaptability.

## 5. Optimization-Based Planning Algorithms

The optimization-based trajectory planning method aims to solve for the optimal trajectory by maximizing or minimizing specific metrics under certain constraints [68]. In realizing this process, Model Predictive Control, as an advanced control strategy, is often used to optimize and track trajectories, ensuring the safety and efficiency of vehicles or robots in dynamic environments [69]. The Artificial Potential Field method, on the other hand, is another commonly used optimization-based trajectory planning method. It guides vehicles or robots to avoid obstacles and move toward the target by constructing virtual attractive and repulsive fields [70].

### 5.1. Model Predictive Control (MPC)

**Figure 9:**
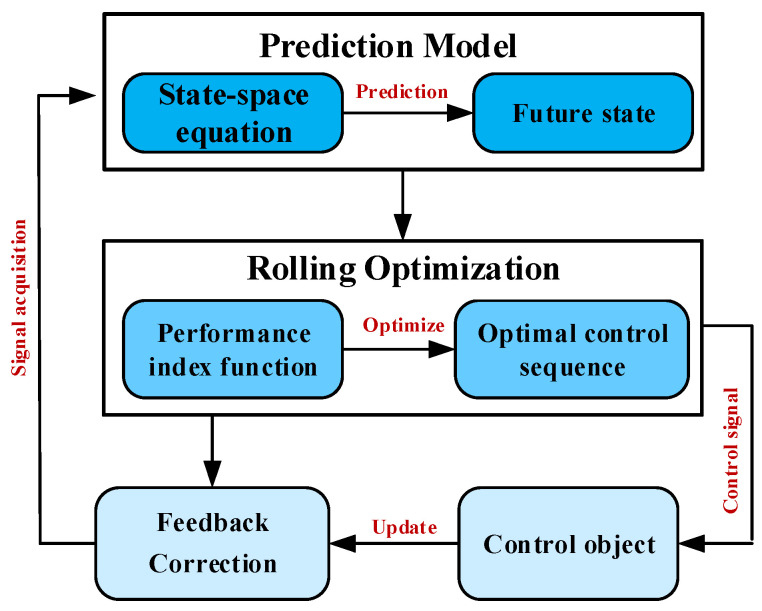
MPC framework.

MPC is an advanced control strategy that integrates strategies such as prediction models, rolling optimization, and feedback correction [71]. As shown in Figure 9, MPC predicts the future behavior of the system over a certain time horizon based on the current system state and environmental information. Based on this prediction, it optimizes the control inputs in order to achieve the optimal performance index (such as total time, energy consumption, safety, etc.) within a future time window [72].

MPC is a powerful control algorithm suitable for scenarios requiring high-precision control and handling of multiple constraints [73]. However, its high computational complexity, model dependency, sensitivity to noise and disturbances, as well as difficulties in parameter tuning, limit its application in real-time and complex environments. By improving the model and introducing distributed architectures, these limitations can be overcome to a certain extent.

#### 5.1.1. Improving Model

Batkovic et al. [74] effectively addressed the shortcomings of traditional MPC algorithms in terms of safety by introducing a safe terminal set, constructing predictive models for unknown constraints, and employing a flexible trajectory tracking control framework. Chen et al. [75] designed an MPC system that incorporates pedestrian path prediction results. This system utilizes the “bicycle model” for dynamic modeling. The objective function is crafted to take into account multiple aspects, such as avoiding pedestrians, tracking the reference path, and ensuring path smoothness. The pedestrian path prediction results, obtained by combining Att-LSTM and MSFM, serve as inputs to the MPC system.

#### 5.1.2. Introduction of Layered Architecture

Shi et al. [76] constructed a hierarchical architecture for trajectory planning and control. The high-level planner considers vehicle kinematics and collision constraints, employing a kinematic model of the vehicle. It generates collision-free trajectories by minimizing a cost function and convexifies non-convex regions to ensure safety. The low-level controller takes into account the vehicle’s lateral and yaw dynamics, adopting a dynamic bicycle model. It receives trajectories generated by the high-level planner and computes optimal control inputs using MPC to achieve stable tracking while defining critical regions around obstacles to prevent collisions. Wang et al. [77] designed a hierarchical planning framework. In the global planning stage, the A* algorithm is employed to generate the shortest path, and a virtual target (VT) is set to move along this path. In the local planning stage, the robot pursues the VT and tracks the global path, generating local paths through the MPC framework. The prediction horizon of the MPC is adaptively adjusted based on the degree of environmental congestion. Additionally, an event-triggered mechanism is developed to determine the execution frequency of the MPC by accumulating control errors. When the distance between the robot and the VT exceeds a certain threshold, the global path is re-planned to take into account the presence of dynamic obstacles.

### 5.2. Artificial Potential Field (APF)

**Figure 10:**
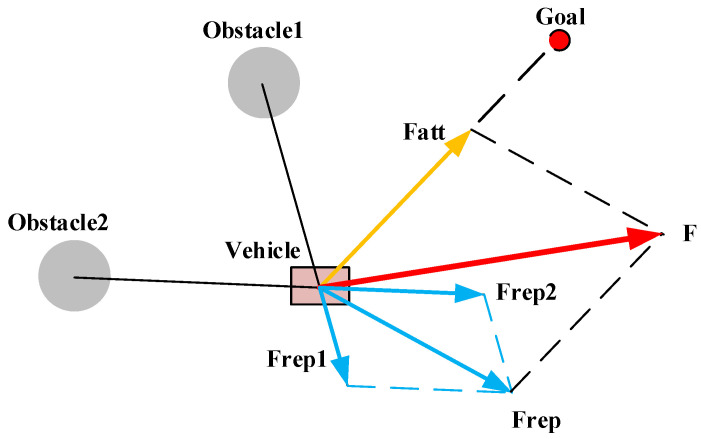
Force condition of a vehicle in a potential field.

APF is a path-planning method based on a virtual force field. As shown in Figure 10, it treats the target location as a source of attraction, generating a force that pulls the vehicle towards the target. At the same time, it considers obstacles as sources of repulsion, producing a force that pushes the vehicle away from them. Under the combined influence of attraction and repulsion, the vehicle moves in the direction of decreasing potential field value, thereby planning a collision-free trajectory [78].

APF has advantages such as easy mathematical expression and simple computation, allowing for an intuitive description of the relationship between the vehicle, its environment, and obstacles. However, this method also has some drawbacks, such as being prone to falling into local minima and producing unsatisfactory planning results in complex environments [79]. By improving the repulsive force function, refining the potential field distribution, and integrating other path-planning algorithms, it is possible to effectively overcome these limitations and enhance the performance and applicability of the APF algorithm.

#### 5.2.1. Improving the Repulsive Force Function

Fan et al. [80] improved the APF method by incorporating a distance correction factor into the repulsive field function, ensuring that the attractive force dominates near the target point. They introduced the Regular Hexagon Guidance (RHG) method, where the robot moves along the edges of a virtual regular hexagon to escape local minimum regions. Additionally, they proposed a relative velocity method to handle dynamic obstacles, adjusting the obstacle avoidance strategy based on the relative speed and direction of the obstacles to ensure effective navigation for the robot in complex environments. Tian et al. [81] enhanced the APF method by adjusting the gravitational field to avoid initial strong pulls towards obstacles, refining the repulsive field with an adjustment factor to mitigate oscillations near the target, introducing a road boundary potential field to constrain driving, and developing a strategy to escape local minima. When trapped, the vehicle searches for escape points within its steering angle range and chooses the one with the lowest potential field value as its new direction.

#### 5.2.2. Improving the Potential Field Distribution

Liu et al. [82] proposed the TPFF-APPS system. This system designs a velocity potential field for dynamic obstacles, dynamically adjusting the path using velocity information. By integrating the velocity potential field with the traditional APF, a comprehensive potential field is formed. The system employs a fuzzy reasoning algorithm to dynamically allocate weights to the two potential fields based on parameters such as predicted collision time, enhancing the flexibility and safety of path planning. Zhang et al. [83] developed an enhanced APF approach that incorporates road and velocity repulsion fields. It dynamically adjusts the magnitude or range of the repulsion field based on the speed of obstacles and includes a target distance factor to ensure that the resultant force is zero when the vehicle reaches the target point. When the vehicle stagnates because of multiple repulsive forces, a virtual sub-target point is randomly generated near the target point to guide the vehicle away from the local minimum region.

#### 5.2.3. Integrating Other Path Planning Algorithms

Chen et al. [84] combined a three-neighbor search A* algorithm with a partial artificial potential field for path planning. The A* algorithm searches only three adjacent nodes along the target direction, reducing redundancy. Using the gravitational field of the potential field enables quick planning in obstacle-free zones. When near obstacles, it switches to the three-neighbor search A* algorithm for precise avoidance, ensuring speed and increasing the chance of finding the best path. Sang et al. [85] proposed the Multiple Sub-target Artificial Potential Field (MTAPF) algorithm. This algorithm improves the A* by reducing search points near obstacles, setting constraints on maximum search distance and path length, incorporating turning costs, and applying trajectory optimization algorithms to generate a globally optimal path. In local path planning, the MTAPF algorithm divides this path into a sequence of multiple sub-target points, allowing the system to switch to the next sub-target point when trapped in a local minimum. Additionally, it converts attractive and repulsive forces into potential field intensities, thereby enhancing the stability and accuracy of path planning. More studies are shown in Table 7.

### 5.3. Summary

In summary, researchers have effectively reduced the computational burden of MPC and APF and improved their real-time performance through algorithm optimization, parallel computing technology, and obstacle model optimization. At the same time, adaptive models, intelligent parameter adjustment, and adaptive potential field distribution methods have been proposed for parameter settings, simplifying the process and enhancing the flexibility and adaptability of the algorithms. Despite these improvements significantly enhancing the performance of MPC and APF methods, some common issues may still persist. For example, MPC suffers from model dependency, which may lead to poor control performance when there is a deviation between the model and the actual system. On the other hand, the parameter settings for the attractive and repulsive fields in APF are relatively complex, and improper settings can affect the accuracy and efficiency of robot path planning.

## 6. Intelligent Algorithms

In path planning, intelligent algorithms play a crucial role, especially when dealing with complex, dynamic, or large-scale environments [90]. These algorithms seek to find optimal or near-optimal paths by simulating natural processes or utilizing advanced mathematical and statistical methods [91]. Common intelligent algorithms include genetic algorithms, particle swarm optimization, ant colony optimization, deep learning, reinforcement learning, deep reinforcement learning, and so on.

### 6.1. Genetic Algorithm (GA)

**Figure 11:**
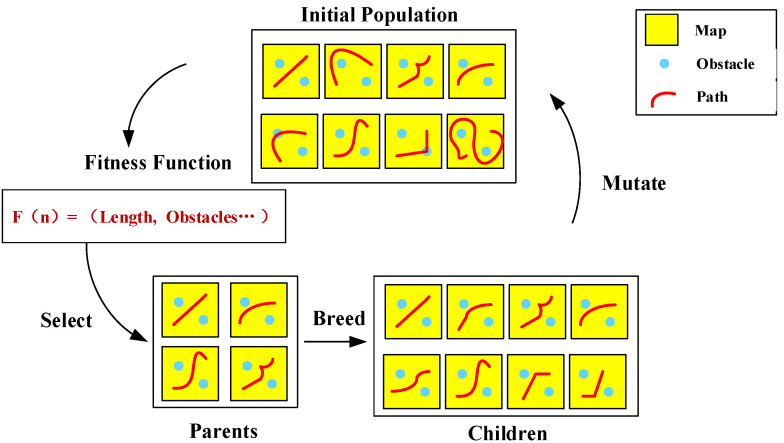
One Generation.

The path planning optimization algorithm based on the genetic algorithm is a heuristic optimization method that seeks to find the optimal path by simulating natural selection and genetic mechanisms, as shown in Figure 11. In the problem of path planning, each individual can be represented as a path consisting of a series of nodes or coordinate points. Each node represents a location or action along the path. The fitness of each individual (path) is evaluated to assess its performance in the solution space. In path planning, the fitness function is usually measured based on indicators such as the length of the path, the number of cells the path passes through, and the ability to avoid obstacles. New individuals are generated through genetic operations, and a portion of them are selected for retention as the parents of the next generation based on fitness evaluation. This process is repeated until a stopping condition is met, such as reaching the maximum number of iterations or finding a satisfactory solution. The path planning optimization algorithm based on the genetic algorithm can efficiently search through complex path spaces to find the optimal path solution that satisfies specific optimization goals [92,93].

The application of Genetic Algorithm (GA) in path planning boasts advantages such as strong global search capability and good robustness, but it also exhibits notable drawbacks, including dependence on the initial population, slow convergence speed, and weak local search ability. To address these shortcomings, researchers have proposed various improvement methods.

#### 6.1.1. Improving the Initial Population

Li et al. [94] proposed the IMGA algorithm. This algorithm randomly generates initial nodes and connects them using the median insertion method to improve the quality of the initial population. A multi-objective fitness function is established by considering path length, safety, and energy consumption. The hierarchical selection method is adopted to maintain population diversity. New populations are generated through single-point crossover and eight-neighborhood single-point mutation. Finally, redundant nodes are removed to reduce turns and shorten path length, thereby enhancing the planning effect. Hao et al. [95] proposed a Multi-Population Migration Genetic Algorithm (MPMGA), which randomly divides a large population into several smaller populations. Each smaller population performs genetic algorithm operations, and information exchange among individuals is achieved through a migration mechanism between populations. During the migration process, high-quality individuals are moved to high-performance populations based on their fitness, while low-quality individuals are removed from these high-performance populations, thereby optimizing the populations. Through migration operations, MPMGA achieves collaborative evolution among multiple populations, jointly driving the algorithm towards finding the global optimal solution.

#### 6.1.2. Improving Convergence Speed

Hao et al. [96] proposed an Adaptive Genetic Algorithm Based on Collision Detection (AGACD). This algorithm employs prior knowledge and random perturbation methods to initialize the population. It incorporates the Delphi weighting method to determine the weights of the fitness function. By combining the roulette wheel selection with elitist selection strategies, through dynamically adjusting the crossover and mutation probabilities, introducing a collision detection algorithm, and utilizing a path optimization operator, the algorithm enhances its convergence speed, path quality, and real-time performance.

#### 6.1.3. Enhancing Local Search Capability

Receveur et al. [97] proposed a new method that combines GA with Fractional Potential Fields (PF). GA generates an initial population, where each individual represents a trajectory. Through an evaluation by a fitness function, which comprehensively considers five objectives, including trajectory length, time, etc., and iterative optimization via genetic operations, the globally optimal path is obtained. PF is utilized for local planning and obstacle avoidance, generating potential fields of different orders based on the characteristics of obstacles and redefining the attractive potential field as a controller to generate dynamic target points. During driving, PF is employed to adjust the trajectory in real-time, achieving obstacle avoidance.

#### 6.1.4. Hybrid Algorithm

Additionally, the hybridization of GA with other algorithms can also improve the efficiency and quality of path planning.

Wang et al. [98] combined APF with GA for path planning. This approach quantifies the environment by using a grid method. By leveraging APF, potential field models are constructed, encompassing road boundary lines, road divider lines, environmental vehicles, and target points. Subsequently, the next step’s path is selected based on the resultant force acting on the vehicle and the orientation of the grid, generating a set of continuous and obstacle-free initial paths that serve as the initial population for GA. The reciprocal of the path length is used as the fitness function, and through iterative optimization of the population via genetic operations; the globally optimal path is ultimately identified. Wang et al. [99] combined MPC with GA for path planning. This method randomly generates an initial population of solutions, where each solution represents a sequence of control inputs. For each solution, the MPC model is used to predict the system’s future behavior, and a fitness value is calculated. Based on the fitness values, superior solutions are selected as parents. These parent solutions then undergo crossover and mutation operations to generate a population of offspring solutions. The above process is repeated until the termination conditions are met. More studies are shown in Table 8.

### 6.2. Particle Swarm Optimization (PSO)

**Figure 12:**
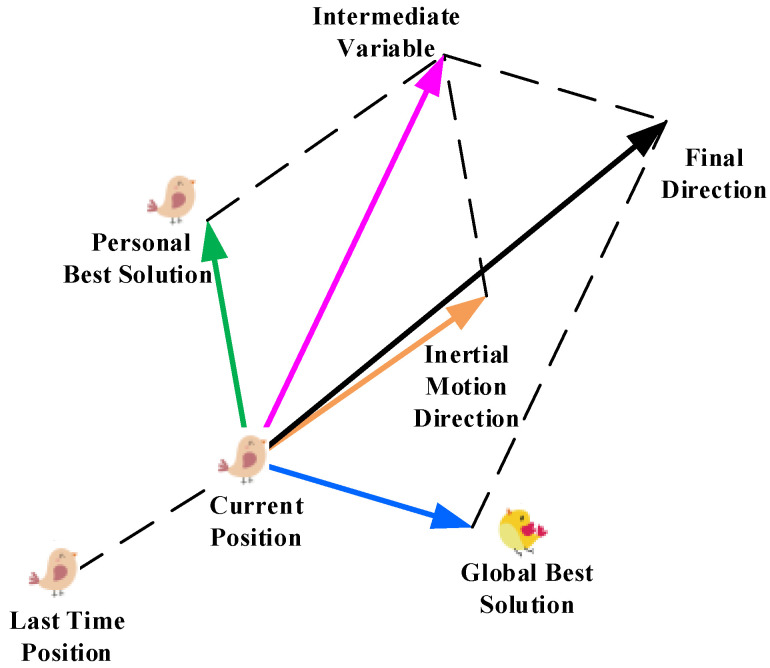
PSO Algorithm.

PSO is an optimization algorithm based on swarm intelligence, which simulates the behavior of bird flocks or fish schools in the process of searching for food [102]. As shown in Figure 12, each individual (called a particle) represents a potential solution, and the swarm of particles collaboratively adjusts their positions based on mutual cooperation and individual experiences to gradually find the global optimal solution [103,104].

In the path planning problem, the PSO algorithm first randomly initializes a set of particles in the search space, where each particle represents a potential path planning solution (i.e., a set of path points). The fitness value is calculated for each particle’s position (i.e., the path planning solution), and this fitness value typically represents the total length or total cost of the path while also taking into account the need to avoid obstacles. For each particle, compare its current solution with its personal best solution. If the current solution is better, update the personal best solution. At the same time, compare the personal best solutions of all particles to find the global best solution. Update the velocity and position of each particle based on its personal best solution and the global best solution so that the particles move towards better solutions [105].

PSO algorithm exhibits excellent performance in path planning, but it may also suffer from issues such as premature convergence and the curse of dimensionality [106]. Researchers have proposed various improved methods to address these challenges.

Chu et al. [107] proposed an improved PSO that generates an initial swarm using an enhanced logistic chaotic map and dynamically adjusts the inertia weight. It maintains velocity direction for improved fitness and adjusts otherwise. To escape local optima, it perturbs the global best particle’s velocity. The logistic chaotic map mutates the global best during iterations, preventing early convergence. Thammachantuek et al. [108] proposed MOEPSO, a PSO-based algorithm that optimizes path length, smoothness, and safety using Pareto optimality. It maintains a Pareto front of non-dominated solutions, updating this front with better solutions as they are found. Upon termination, the Pareto front provides an approximate optimal solution set. There are also some hybrid algorithms that can improve upon the limitations of the PSO, as shown in Table 9.

### 6.3. Ant Colony Optimization (ACO)

**Figure 13:**
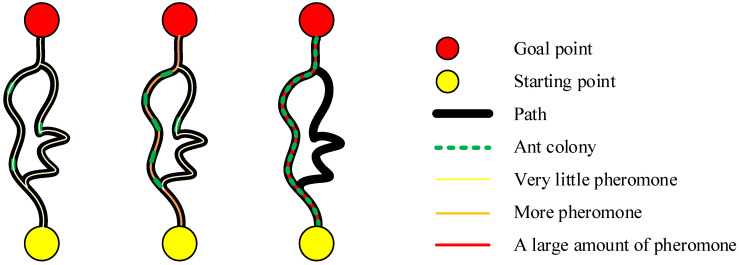
ACO Algorithm.

The fundamental principle of the Ant Colony Algorithm lies in simulating the pheromone release and path selection mechanisms observed in ants during their foraging process [117]. Ants leave pheromones as they walk, and subsequent ants tend to follow paths with higher concentrations of pheromones. Over time, the concentration of pheromones on good paths increases due to the travel of more ants, which attracts even more ants, forming a positive feedback mechanism that gradually identifies and reinforces the optimal path [118], as shown in Figure 13.

In path planning, the initial step is to initialize the pheromone concentration on all possible paths. Each ant starts from the initial node and independently selects the next node based on the pheromone concentration and heuristic information (such as the reciprocal of path length) on each path. The selection probability for each path is proportional to its pheromone concentration and heuristic information, and a path is randomly chosen according to these probabilities. As ants traverse the path, they release pheromones, with the amount released being influenced by factors such as the path length. During the iteration process, the pheromone concentration on shorter paths gradually accumulates, attracting more ants to choose this path and thereby gradually approaching the optimal path. Upon completion of the iterations, the algorithm selects the path with the highest pheromone concentration, which is then output as the optimal path [119,120].

Despite the significant achievements of ACO in solving path planning problems, it still exhibits certain limitations, such as a lack of initial pheromones, slow convergence speed, and a tendency to fall into local optima. Consequently, various improvement methods have been developed.

#### 6.3.1. Addressing the Issue of Initial Pheromone Deficiency

Wang et al. [121] proposed a multi-factor path planning method based on the ant colony algorithm. This method integrates fuzzy mathematics and the Analytic Hierarchy Process (AHP) to construct an evaluation system, comprehensively assessing environmental factors and their weights. By introducing the expected inverse distance from nodes to the target point, the probability formula for ants to select subsequent nodes is optimized. Additionally, parameters related to inflection points and the sum of weights are incorporated into the pheromone update process, guiding ants to choose shorter paths with less negative environmental impact.

#### 6.3.2. Addressing the Issue of Slow Convergence

Xiong et al. [122] made four improvements to the ACO algorithm: First, they proposed a time taboo strategy, prohibiting ants from visiting certain nodes during specific time periods to avoid redundancy and collisions. Second, they designed a three-step arbitration method to temporarily prohibit access to nodes likely to cause redundancy, thus accelerating convergence. Third, they employed a rollback strategy, enabling ants to backtrack two steps when encountering deadlocks or self-locks. Fourth, they constructed an occupancy grid prediction model based on the motion model and predicted angles of dynamic obstacles to forecast their future positions. Ajeil et al. [123] proposed an Age-Based Aging ACO (ABACO) algorithm: by taking into account the age of ants to control the amount of pheromone released, younger ants (i.e., ants with shorter paths) release more pheromones, thereby accelerating the convergence of the algorithm. Combined with a time taboo strategy, a three-step arbitration method, and an occupancy grid prediction model, the algorithm’s path planning performance in both static and dynamic environments is significantly improved.

#### 6.3.3. Addressing the Issue of Local Optima

Wang et al. [124] proposed an improved ACO algorithm. This algorithm generates the shortest path using the Floyd algorithm and sets a high initial pheromone concentration. Subsequently, the ant colony searches for paths based on the heuristic function and pheromone levels. When encountering a deadlock, a backtracking strategy is implemented, adding the current node to the taboo list and returning to the previous node while reducing the pheromone concentration at the current node. During iterations, pheromones are dynamically updated according to a multi-objective optimization function. Finally, the path undergoes connectivity processing and quadratic B-spline curve optimization.

#### 6.3.4. Hybrid Algorithm

Additionally, there are hybrid algorithms that have also improved upon the limitations of ACO, as shown in Table 10.

### 6.4. Deep Learning (DL)

DL is a machine learning method in the field of artificial intelligence. It automatically learns and extracts features from large amounts of data by constructing and training multi-layer neural network models, thereby enabling automated processing and decision-making for complex tasks.

Graph Neural Networks (GNNs) are an important branch in the field of deep learning. GNNs can serve as a component or technical framework within path planning algorithms, used to process graph-structured data and extract features. They are typically not used as a standalone path planning algorithm but rather combined with other algorithms to jointly solve path planning problems.

**Figure 14:**
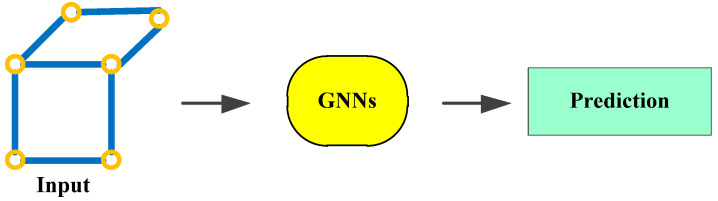
GNNs.

As shown in Figure 14, the actual scene is first abstracted into a graph structure, where nodes represent key locations or entities, and edges represent the connection relationships between them. Next, corresponding features such as position and speed are assigned to each node and edge in the graph. Then, a suitable GNN model is designed to extract high-level features from the graph through multiple layers of graph convolution and output the path selection probability for each node. Finally, based on the prediction results of the GNN model, relevant algorithms are used to generate the optimal path from the starting point to the destination.

Table 11 presents some research on path planning that combines GNN with other algorithms.

### 6.5. Reinforcement Learning (RL)

**Figure 15:**
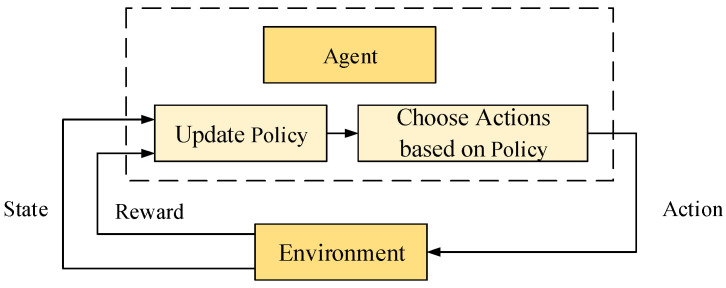
RL System.

In path planning, RL enables agents to learn how to navigate complex environments through repeated trials and learning [136]. The agent selects actions (such as movement directions or strategies) based on its current state (location and environmental information), thereby influencing its position changes in the environment and receiving rewards (such as shorter paths or successful obstacle avoidance). Through interactions with the environment, the agent gradually learns the optimal strategy to complete the planning task [137], as shown in Figure 15.

**Figure 16:**
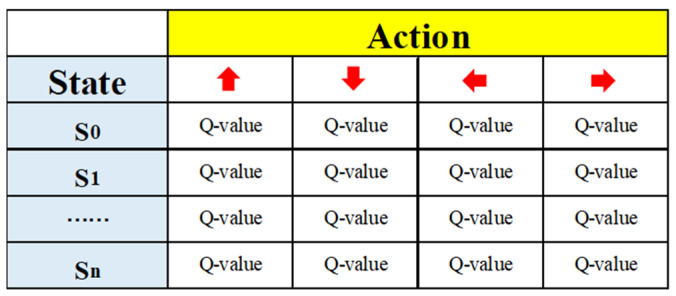
Q-Table.

Q-learning is a classic algorithm in reinforcement learning that learns the optimal strategy by estimating the long-term return value (Q-value) for each state-action pair [138]. Figure 16 presents a Q-value table, where the rows represent different states (such as S_0_ to S_n_), and the columns represent different actions (indicated by up, down, left, and right arrows). When in a certain state, the agent refers to the Q-value table and selects the action with the highest Q-value to execute. The environment then provides a reward based on the agent’s action and updates the agent’s state. This process continues in a loop, with the agent gradually optimizing the Q-values to learn the optimal strategy [139]. However, the traditional Q-learning algorithm suffers from slow convergence and requires a large number of iterations in complex environments [140]. In order to enhance the efficiency of path planning and accelerate convergence, many scholars have made improvements to it.

Ma et al. [141] proposed an improved Q-learning algorithm (CLSQL) based on a continuous local search strategy. This algorithm gradually divides the global environment into independent local environments. In each local environment, the Euclidean distance function is utilized to establish prior knowledge of the environment, optimizing the Q-table and achieving local optimization. Subsequently, a global path is formed by connecting the optimal paths in each local environment. Finally, the Q-table is updated based on the movement results and the reward function until the iteration process concludes. Zhao et al. [142] proposed the EMQL algorithm, an improved Q-learning algorithm based on experience memory. This algorithm optimizes in four aspects: it combines static and dynamic rewards to guide robot exploration, introduces an experience memory table (EM table) to record the shortest path for improving convergence speed, utilizes an instruction table to store path nodes for guiding robot movement, and leverages the Q-table and EM table to quickly replan paths in case of destination changes or path obstructions. Shi et al. [143] proposed an improved RL algorithm. They initialized Q-values based on the distance from each state to the goal position, assigning higher Q-values to states closer to the goal to guide the robot toward the goal in the early stages. The algorithm also dynamically adjusts the ε-greedy factor: a larger ε in the initial stages encourages exploration, while a gradually decreasing ε in later stages enhances exploitation.

More studies are shown in Table 12.

### 6.6. Deep Reinforcement Learning (DRL)

DRL is a machine learning approach that combines deep learning with RL, leveraging deep neural networks to process high-dimensional and complex state spaces, thereby achieving the goals of reinforcement learning [136].

**Figure 17:**
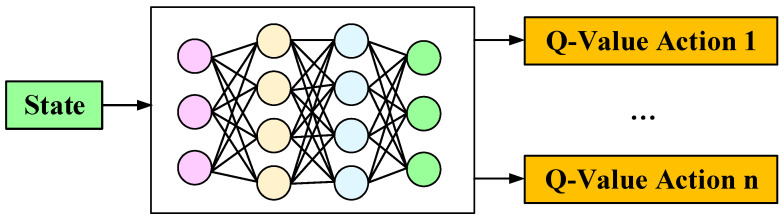
DQN.

Deep Q-Network (DQN) is a specific algorithm in DRLn [148]. The DQN first initializes the environment settings and network parameters. Then, the agent explores the environment randomly to collect empirical data. By utilizing the experience replay mechanism, it randomly samples these data to stabilize the training process. Next, the algorithm trains the neural network (which serves as the Q-network) to evaluate the value of actions by calculating the target Q-values. This process is iterated continuously, with the network parameters being updated until the training requirements are met [149], as shown in Figure 17. However, DQN also faces issues such as slow convergence and low learning efficiency.

#### 6.6.1. Regarding the Slow Convergence Speed

Han et al. [150] proposed an improved DDQN method. It discretizes high-dimensional LiDAR data and uses a dual-branch network for navigation and obstacle avoidance. An “expert experience” module accelerates convergence, while “reward shaping” ensures timely feedback, addressing sparse rewards. Ren et al. [151] proposed a two-stage DRL method that combines Dynamic Programming (DP) and Extreme Learning Machine (ELM). First, the DP method is used to map the path planning problem, find the global optimal solution, and generate high-quality training data. Subsequently, ELM is employed to rapidly initialize the parameters of the actor and critic neural networks in DRL, bringing them close to the optimal solution. Finally, fine-tuning is carried out using a modified Deep Deterministic Policy Gradient (DDPG) method to ensure high accuracy. Zheng et al. [152] enhanced the DQN algorithm. They utilized a PTZ camera for symmetric environment images and target location as neural network inputs. New reward points were introduced with dynamic adjustments based on relative positions. Target points for the patrol robot were dynamically calculated. The neural network was adjusted to a mixed architecture with convolutional and fully connected layers, incorporating experience replay and target network mechanisms.

#### 6.6.2. Regarding the Low Sample Efficiency

Zhang et al. [153] made improvements to the experience replay buffer and reward function in DQN. To address the issue of high sample similarity in the experience replay buffer, they designed a similarity screening matrix. Before each extraction of samples for training, this matrix is used to identify and remove highly similar samples, thereby enhancing the quality of these training data. Additionally, the improved reward function not only takes into account the basic reward for reaching the destination but also incorporates a dynamic reward adjustment mechanism related to path length and obstacle avoidance.

#### 6.6.3. Hybrid Algorithm

By combining different algorithms with DQN, the performance of the algorithm can be improved. As shown in Table 13.

### 6.7. Summary

The improvement methods for intelligent algorithms are diverse, such as integrating multiple algorithms, refining parameter update mechanisms, and introducing new operators. However, these improved algorithms may still have some common defects, such as high computational resource requirements and insufficient generalization ability for complex problems.

## 7. Conclusions

Path planning is a core function of autonomous mobile robot technology. Its primary tasks include rapidly planning a collision-free path based on a global map and locally and dynamically adjusting this global path according to real-time environmental information. This paper reviews 15 common path-planning algorithms, discussing their advantages, disadvantages, and current development status.

Among the 15 algorithms discussed in this paper, both graph search-based and sampling-based planning algorithms fall under the category of global planning algorithms. Graph search-based algorithms, such as Dijkstra’s and A*, typically exhibit good completeness, meaning that if a feasible path exists, the algorithm will eventually find it. However, these algorithms are computationally intensive when dealing with large maps and perform poorly in dynamic environments. Sampling-based planning algorithms, such as PRM and RRT, are more computationally efficient and can adapt to dynamic changes, but they do not guarantee finding the optimal path. The initial paths generated by both types of algorithms are not smooth and require subsequent processing.

Both geometric curve-based planning algorithms and optimization-based planning algorithms belong to local planning algorithms. Geometric curve-based planning methods are computationally simple, and the curves can be adjusted and modified as needed. However, they struggle with handling complex environments. Optimization-based planning methods, such as MPC and APF, can deal with various complex environments and dynamic changes, but they have high computational complexity, strong model dependency, and challenging debugging processes.

Intelligent algorithms are capable of both global and local planning. GA possesses strong global search capabilities and can adapt to complex environments, but it is complex to program, has slow search speeds, and may converge too early. PSO algorithms are simple to implement and have fast convergence speeds, but they may fall into local optimal solutions. ACO algorithms are robust, but they are computationally intensive, prone to falling into local optima, and have slow convergence speeds. DL has relatively weak capabilities in independent path planning and needs to be used in conjunction with other algorithms. RL excels in learning ability and adapting to complex environments, but it requires long training times and has low exploration efficiency. DRL can handle complex problems, but it has high training costs and convergence difficulties.

**Figure 18:**
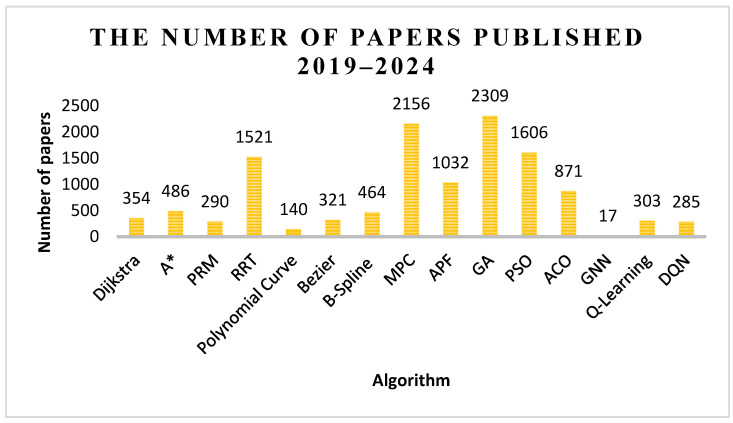
The number of papers on various algorithms.

Researchers have conducted extensive work on optimizing these algorithms. Figure 18 shows the number of papers on various path-planning algorithms catalogued on Web of Science between 2019 and 2024.

Based on data shown in Figure 18, there are significant differences in the number of publications for various algorithms.

The GA tops the list with 2309 papers, which, to some extent, is attributed to its significant advantages in tackling complex problems. MPC follows closely with 2156 papers. This is largely because these two algorithms can effectively solve complex problems in multiple fields (such as industrial control, robotics, etc.) and demonstrate broad application potential. As a result, they have attracted researchers from different fields to conduct in-depth research and application expansion, gradually forming a relatively complete theoretical foundation and algorithmic framework. This provides certain theoretical support for researchers to apply and improve GA and MPC in the field of path planning, lowers the research threshold, and thus stimulates the interest of more people to conduct related research, leading to the publication of a large number of related papers.

Due to the good applicability of PSO in global path exploration and the excellent performance of RRT in dynamic programming scenarios, they have received attention from 1606 and 1521 papers, respectively. This may be because the applications of these two algorithms are more focused on certain specific fields or problems: PSO is primarily applied to traditional optimization problems and specific intelligent control areas, while RRT is more frequently used in robot motion planning and autonomous driving, among others. Compared to the broad fields covered by GA and MPC, the research popularity and application scope of these scenarios may be relatively limited; thus, the number of papers may be slightly less than that of GA and MPC. However, it is worth noting that the research depth and breadth of PSO and RRT are continuously expanding.

Moreover, APF and ACO have shown promising performance in addressing local path planning problems, with 1032 and 871 papers published on them, respectively. This may be due to the fact that the fundamental theory of the APF algorithm is relatively mature, making it somewhat challenging to achieve significant innovations within the existing theoretical framework. Consequently, the growth of new research findings and the number of papers may be relatively slow. On the other hand, the inherent complexity and parameter sensitivity of the ACO algorithm often require more in-depth theoretical analysis and extensive experimental validation for optimization and improvement. This may make high-quality research outcomes related to ACO relatively less accessible, thereby limiting the number of papers to some extent.

Compared with other algorithms, despite their classic status, Dijkstra’s algorithm and A* algorithm have relatively few papers published, with 354 and 486 papers, respectively. This might be because these two algorithms have been around for a long time since they were proposed, and their fundamental principles and core technologies have become relatively mature and stable. Therefore, research papers solely focused on the principles of these two algorithms may be relatively scarce. Nevertheless, these two algorithms still play a very important role in practical applications. Dijkstra’s algorithm is often one of the preferred algorithms in network routing, map navigation, and logistics distribution systems. A* algorithm, on the other hand, has been widely applied in fields such as robot navigation, game development, and autonomous driving. Furthermore, Dijkstra’s algorithm and A* algorithm have also provided valuable insights and foundations for the development of other path-planning algorithms.

The polynomial curves, Bezier curves, and B-spline curves have also received a certain amount of attention, with 140, 321, and 464 papers published, respectively. Similar to Dijkstra’s algorithm and the A* algorithm, these three types of curves, as commonly used methods for path smoothing, have relatively mature fundamental principles and algorithmic frameworks and are more focused on practical applications. Therefore, the number of research papers on them may be relatively less. The research on these curves themselves might be approaching a certain level of saturation, with new research increasingly focusing on the optimization, improvement of the curves, and their integrated application with other technologies.

PRM has only 290 papers, perhaps because its principles are relatively simple and its core idea is quite straightforward, leaving not a great deal of room for in-depth exploration and innovation. At the pure algorithmic principle level, the extensible research content may be relatively limited, so the number of research papers based on the algorithm itself may not be too high. In addition, PRM is mainly applied in specific fields, such as robot path planning and motion planning in computer graphics. In many other engineering and scientific fields, its application is not particularly widespread, which may limit its application scenarios to some extent, and relatively few people may pay attention to or research it, naturally affecting the number of papers.

The algorithms in the field of machine learning, such as GNN, Q-learning, and DQN, although relatively new, are gradually attracting the attention of researchers, with 17, 303, and 285 papers published, respectively. This may suggest that these algorithms still have some issues to be explored and resolved, as well as areas that need further refinement and improvement. Researchers have conducted in-depth discussions and studies on the theoretical foundations, mathematical models, and operational mechanisms of these algorithms through publishing papers, hoping to address the existing problems, further refine their theoretical frameworks, and make these algorithms more mature and reliable.

Specifically, intelligent algorithms and optimization-based algorithms are gradually gaining attention and becoming hot trends in path-planning research and applications. Meanwhile, the influence of classic algorithms remains significant. They often play a role as fundamental or submodule components in the improvement frameworks of new algorithms or are directly applied to solve practical problems.

In addition to the papers published by researchers, many open-source autonomous driving software programs have also integrated various path-planning algorithms, providing powerful path-planning capabilities for autonomous vehicles, as shown in Table 14. This open-source software not only facilitates the popularization and advancement of autonomous driving technology but also enables developers to more conveniently implement and test various path planning algorithms, thereby driving continuous innovation and development of the technology.

In summary, the field of path planning is undergoing a transition from traditional algorithms to intelligent and optimization-based algorithms, with various algorithms demonstrating unique advantages in specific application scenarios. From classic algorithms such as A* and Dijkstra to modern intelligent algorithms such as DQN and PSO, diverse path-planning methods provide a range of solutions for the development of autonomous mobile robot technology. At the same time, the widespread use of open-source platforms has lowered the technical barriers, offering researchers and developers efficient testing environments and opportunities for algorithm innovation. In the future, with the enhancement of computing capabilities and advancements in deep learning, path-planning algorithms will become more intelligent and adaptive, enabling safer and more efficient driving in complex and dynamic traffic environments. The continuous progress in technology also implies that multi-algorithm fusion and collaborative optimization will emerge as new trends, laying a more solid foundation for the maturity and large-scale application of autonomous driving systems.

## Figures and Tables

**Table 1:** Limitations and improvement measures of A*.

Ref.	Disadvantage	Improvement	Method Description
[16,17,18,19,20,21]	Excessive redundant nodes	Optimization of the heuristic function	Construct a new evaluation function to reduce the number of nodes during the search.
[16,17,22,23]	Slow planning speed	Bidirectional search	Conduct searches simultaneously from both the starting point and the endpoint.
[16,19,22]	Excessive inflection points	Path smoothing	Reduce the number of turning points and right-angle turns.
[21,23,24,25]	Excessive inflection points	Hybrid algorithm	Combining polynomial curves, Bezier curves-spline curves for smooth path planning.
[16,22]	High collisionrisk	Extended distance	Expand a certain distance outward from the edge of obstacles to form a safety buffer.

**Table 2:** The comparison between Dijkstra and A*.

Comparison	Dijkstra	A*
**Search Method**	Only considers path cost	Combines path cost and heuristic function
**Heuristic Information**	No heuristic information	Requires a heuristic function h(n)
**Application**	Network routing	Autonomous driving
**Memory Consumption**	High (traverse all nodes)	High (maintain open list and closed list)
**Optimality**	Find the optimal solution	Can find the optimal solution with suitable h(n)
**Dynamic Environments**	Not suitable	Not suitable
**Computational Efficiency**	Low (needs to traverse all paths)	High (when optimized by the heuristic function)

**Table 3:** The hybridization of PRM with other algorithms.

Ref.	Hybrid	Solved Problem	Method Description
[39]	D*	Improve search efficiency	The D* algorithm is employed to conduct a secondary search for nodes that cannot be directly connected in the PRM construction phase.
[40]	A*	Improve search efficiency while managing dynamic obstacles	PRM constructs a global roadmap, A* algorithm is used for local search.
[41]	APF	Increase the density of sampling points in narrow passages	Using PRM for global planning, the Artificial Potential Field is used to optimize obstacle points.

**Table 4:** The hybridization of RRT with other algorithms.

Ref.	Hybrid	Solved Problem	Method Description
[46]	GWO	Shorten path length	Use Grey Wolf Optimization to determine the optimal length and direction for each movement.
[47]	Dijkstra	Improve search efficiency	Adjustable probability strategies dynamically adjust the target biasing probability based on the state of the search tree. The Dijkstra algorithm is used to prune the initial path and eliminate redundant nodes.
[48]	RPM Dijkstra	Improve path planning efficiency and shorten path length	RPM and RRT each generate a path. Resampling and roadmap construction are carried out within the convex hull area defined by the outer points of the two paths. Finally, Dijkstra’s algorithm is used to find the optimal path.
[49]	A*	Improve convergence speed	RRT generates an initial path, and the A* algorithm is used for local optimization in the area near the initial path.
[50]	APF	Improve search efficiency	Bi-directional RRT generates an initial path, while the Artificial Potential Field method is responsible for real-time obstacle avoidance and trajectory smoothing.

**Table 5:** The comparison between PRM and RRT.

Comparison	PRM	RRT
**Planning method**	Offline roadmap construction	Online real-time expansion
**Environment**	Static environment	Dynamic environment
**Application**	Complex and high-dimensional spaces	Real-time planning and narrow passage
**Path quality**	Better paths	Require post-processing
**Reuse**	Roadmaps can be reused multiple times	The tree needs to be rebuilt for each query
**Narrow passage**	Poor	Good

**Table 6:** Comparison Summary.

Comparison	Polynomial Curves	Bezier Curves	B-Spline
**Definition**	Single polynomial equation	Control points	Control points and knot vector
**Local control**	NO(global changes)	No (global changes)	Yes (only affects the local area)
**Complexity**	Low	Medium	High
**Smoothness**	High-order polynomials may be unstable	Smooth but complex with a large number of control points	Very smooth, supporting high-order continuity
**Application**	Short-range path planning, dynamic constraint control	Local path optimization, high-precision path planning	Complex path planning, parking, and obstacle avoidance

**Table 7:** The hybridization of APF with other algorithms.

Ref.	Hybrid	Improvement	Method Description
[86]	IRRT*	RRT: Introduces adaptive step size and search range, along with path-cutting optimization	For each expanded node, attraction and repulsion functions are constructed to enable the search tree to move more effectively toward the target point under the combined force.
[87]	A*	APF: Adds oscillation detection and local minimum detection A*: Adds local minimum boundary detection	A fusion manager is introduced to receive the results returned by the APF and determine whether to initiate the A* based on those results.
[88]	RRT Bezier	APF: Proposes a heuristic method based on the number of adjacent obstacles RRT: Introduces a triangular nearest neighbor node selection strategy	When the distance to obstacles is greater than twice the step size, the improved APF is used for rapid path expansion; otherwise, the improved RRT is employed for obstacle avoidance, and finally, the path is smoothed using Bezier curves.
[89]	RRT	APF: Improves the force fields RRT: Generates constrained nodes to enhance safety, extracts key nodes to reduce redundancy	The improved RRT is used for global path planning, and the path is divided into multiple sub-path segments. Each sub-path segment is then optimized using the improved APF.

**Table 8:** The hybridization of GA with other algorithms.

Ref.	Hybrid	Improvement	Method Description
[100]	APF	APF: Adopts a time-efficient deterministic approach to optimize potential value assignment and path search processes. GA: Customizes crossover and mutation operators and optimizes path representation and encoding.	Using the improved APF, all initial paths are found and encoded as the initial population. The improved GA is then employed for path optimization.
[101]	GWO	No	GA is used to provide stable initial solutions, while GWO leverages these initial solutions for global optimization.

**Table 9:** The hybridization of PSO with other algorithms.

Ref.	Hybrid	Improvement	Method Description
[109]	APF	APF: Introducing relative velocity and relative acceleration to improve the repulsive force field PSO: Incorporating the Opposing Base Learning strategy, with adaptive adjustment of inertia weight and step size	Using the improved PSO for global path planning and the improved APF for dynamic obstacle avoidance
[110]	HSA	No	PSO conducts a global search, while the Harmony Search Algorithm performs a fine-tuning search around the potential optimal areas identified by PSO
	GA	No	PSO performs a global search, and GA conducts a local search
[111]	GA B-spline	PSO: Adaptive dynamic inertia weight	PSO is responsible for quickly locating potential high-quality solutions in the search space, while GA further optimizes these solutions. Finally, a cubic B-spline curve is used to smooth the path
[112]	DC A*	PSO: Adaptive dynamic inertia weight and chaotic mutation	The optimal solution is selected based on the DC strategy. The A* algorithm is used to generate a path that participates in the evolution process of PSO as a particle
[113]	IDE	PSO: Introducing the concept of corporate governance, optimizing the PSO update formula through adaptive adjustment of weight and acceleration coefficients	The improved PSO is responsible for global search and initial optimization, generating an elite population. Improved Differential Evolution further optimizes and feedback to PSO
[114]	A*	A*: Introducing a strategy for removing redundant nodes PSO: Proposing random inertia weight and random opposing base learning strategy	A* is used to calculate the initial path and key nodes are extracted as the initial particles for the PSO algorithm, which is then optimized using the improved PSO
[115]	SA	PSO: Proposing a global optimal solution update strategy: introducing a dimensional learning strategy	In each iteration, the Simulated Annealing algorithm is used to update the global optimal solution of the PSO algorithm
[116]	Bezier	PSO: Introducing adaptive fractional-order velocity, with dynamic adjustment of acceleration coefficients and inertia weight.	Continuous high-degree Bezier curves are used to generate smooth paths, and the improved PSO algorithm is employed to optimize the control points of these curves

**Table 10:** The hybridization of ACO with other algorithms.

Ref.	Hybrid	Improvement	Method Description
[125]	APF	APF: Optimize the potential field model and construct a dynamic APF gradient ACO: Introduce improved strategies	Using the improved ACO algorithm for global path planning and the enhanced APF algorithm to address collision issues
[126]	APF	APF: Establish a dynamic early warning obstacle avoidance model, redefine the potential field function, and dynamically adjust the step size ACO: Improve the heuristic function and pheromone update rules and introduce a dynamic pheromone evaporation factor	
[127]	GA	GA: Optimize the adjustment of the evaluation function	The initial path generated by ACO is used as the initial population for the GA algorithm
[128]	GA	GA: Introduce the deletion operator to remove unnecessary nodes	
[129]	GA	ACO: Improve the pheromone update rules and transition probability rules and introduce heuristic distance information probability	
[130]	GA	GA: Optimize the fitness function and genetic operation methods. ACO: Optimize the pheromone update strategy and introduce a penalty function to establish a dead-end table.	Utilize multiple optimized paths generated by the GA as the initial pheromones for the ACO.
[131]	RRT	No	A pseudorandom rule determines each iteration’s process: expanding the search tree via RRT for exploration or selecting nodes based on pheromone for exploitation.
[132]	A*	A*: Optimize the heuristic function. ACO: Develop a Representative-Based Estimation (RBE) strategy	ACO determines the target visit order using the cost map, while A* plans the path to each target in that order.

**Table 11:** The hybridization of GNN with other algorithms.

Ref.	Hybrid	Improvement	Method Description
[133]	RL	A hyper-heuristic algorithm is proposed	The heuristic space is parameterized by GNNs
[134]	Convolutional Neural Network (CNN)	GNN: A new reward structure	Features are collected from local observations using CNN, and these data are transmitted among agents by GNN
[135]	The greedy algorithm.	NO	The GNN outputs a set of guidance values for the neighbor set, and then the greedy algorithm is used to select the next vertex based on these guidance values

**Table 12:** The hybridization of Q-Learning with other algorithms.

Ref.	Hybrid	Improvement	Method Description
[144]	APF	Q-Learning: Dynamic Q-Learning	Utilizing APF to initialize the Q-table based on the positional information of obstacles and target points in the environment
[145]	APF	Q-Learning: Introducing APF Weighting in the Decision-Making Process	Selecting the optimal action using Q-values and guiding the robot’s movement direction through APF weighting
[146]	WOA	Q-Learning: New Exploration Strategy with Dynamic Value Adjustment	Employing the Whale Optimization Algorithm (WOA) to generate the Q-table
[147]	A*	A*: Dynamically Adjusting Weights of Actual and Estimated Costs, Introducing Bidirectional Search Strategy Q-Learning: Designing State Space and Action Space, Optimizing Reward Mechanism, Introducing Dynamic Exploration Factor ε	Utilizing an improved A* algorithm for global path planning and an improved Q-learning algorithm for local dynamic path adjustment

**Table 13:** The hybridization of DQN with other algorithms.

Ref.	Hybrid	Improvement	Method Description
[154]	A*,APF	DQN: Proposes an improved ε-greedy strategy and designs a heuristic reward function using A* and APF	Incorporate the improved ε-greedy adaptive exploration strategy and heuristic reward function into the DQN algorithm
[155]	APF B-spline	DQN: A multi-output neural network structure is adopted, and an adaptive SA-ε-greedy algorithm is proposed	Utilize prior knowledge provided by APF to accelerate the DQN learning process and apply cubic B-spline for path smoothing
[156]	APF	DQN: A reward function based on an artificial potential field is introduced	Optimize the training process of DQN by introducing a reward function based on APF
[157]	A*,RRT	DQN: An improved Double DQN.	By integrating the concepts of the A* algorithm and RRT algorithm into the DDQN algorithm, improve the initialization strategy and reward function design of DDQN

**Table 14:** Various autonomous driving software and their path-planning algorithms.

Software	Path Planning Algorithms
Autoware Universe (release/v1.0 beta)	Dijkstra, A*,MPC, Hybrid A*, Frenet Frame, RRT, RRT*, B-Spline, QP (Quadratic Programming), RL, DNN
Apollo v8.0	Dijkstra, A*, Hybrid A*, Frenet Frame, MPC, QP, RPM, RRT, RL, DQN
OpenPilot v0.8.5	Polynomial Path Planning, MPC
ROS 1.0 (Noetic)	It does not directly provide path-planning algorithms but supports the implementation and integration of various path-planning algorithms.
